# Recent Trends in Prostate Cancer Incidence by Age, Cancer Stage, and Grade, the United States, 2001–2007

**DOI:** 10.1155/2012/691380

**Published:** 2012-11-27

**Authors:** Jun Li, Joseph A. Djenaba, Ashwini Soman, Sun Hee Rim, Viraj A. Master

**Affiliations:** ^1^Division of Cancer Prevention and Control, National Center for Chronic Disease Prevention and Health Promotion, CDC, 4770 Buford Hwy, MS K55, Atlanta, GA 30341, USA; ^2^Department of Urology and Winship Cancer Institute, School of Medicine, Emory University, 1365C Clifton Road, Atlanta, GA 30322, USA

## Abstract

*Objective*. To examine prostate cancer trends by demographic and tumor characteristics because a comprehensive examination of recent prostate cancer incidence rates is lacking. *Patients and Methods*. We described prostate cancer incidence rates and trends using the 2001–2007 National Program of Cancer Registries and Surveillance, Epidemiology, and End Results Program data (representing over 93% of US population). Because of coding changes in cancer grade, we restricted analysis to 2004–2007. We conducted descriptive and trend analyses using SEER∗Stat. *Results*. The overall prostate cancer incidence rate was stable from 2001 to 2007; however, rates significantly increased among men aged 40–49 years (APC = 3.0) and decreased among men aged 70–79 years (APC = 2.3), and 80 years or older (APC = −4.4). About 42% of localized prostate cancers diagnosed from 2004 to 2007 were poorly differentiated. The incidence of poorly differentiated cancer significantly increased among localized (APC = 8.0) and regional stage (APC = 6.1) prostate cancers during 2004–2007. *Conclusions*. The recent trend in prostate cancer incidence was stable but varied dramatically by age. Given the large proportion of poorly differentiated disease among localized prostate cancers and its increasing trend in more recent years, continued monitoring of prostate cancer incidence and trends by demographic and tumor characteristics is warranted.

## 1. Introduction

Prostate cancer is the most commonly diagnosed non-skin cancer and the second leading cause of cancer death among American men. Each year, approximately 220,000 men are diagnosed with prostate cancer and 29,000 die from it [1]. With the introduction of the prostate-specific antigen (PSA) testing in the mid-1980s, prostate cancer incidence rate increased drastically, at about 12% per year, and peaked in 1992 [2]. The rate subsequently declined, at about 10% per year for the following three years and then appeared to stabilize from 1995 to 2005 [2, 3]. In 2011, Kohler et al. reported a stable trend of prostate cancer incidence from 1998 to 2007; however, demographic and clinical factors were not examined in this study [4]. With the widespread use of the PSA test, the mean age at diagnosis dropped substantially, from 72.2 years between 1988-1989 to 67.2 years between 2004 and 2005 [5]. Studies using Surveillance, Epidemiology, and End Results Program (SEER) data have shown that the distribution of prostate cancer stage and grade has also dramatically changed, with localized and moderately differentiated tumors becoming predominant [6, 7].

Age at diagnosis, cancer stage, and grade are among the most important factors used to determine the prostate cancer treatment modality such as prostatectomy, radiation, or active surveillance [8]. Since most of screen-detected prostate cancers are low risk and are unlikely to cause death, medical experts have agreed that active surveillance, a way to monitor disease periodically rather than treat it immediately, has emerged as a viable treatment option for patients with low risk prostate cancer (http://consensus.nih.gov/2011/prostate.htm). 

To our knowledge, a comprehensive examination of recent prostate cancer incidence rates and trends in the entire US population is lacking, especially by cancer stage and grade. As the US population ages and life expectancy continue to increase, it is increasingly important to understand distribution and changes in cancer pathological patterns. The purpose of the current study was threefold: first, to describe recent prostate cancer incidence rates and trends by demographic and tumor characteristics using the nationwide cancer registry data; second, to provide age-specific information on pathological patterns of prostate cancer; third, to show distributions and temporal changes of cancer grade within each cancer stage. 

## 2. Patients and Methods

New cases of prostate cancer (incident cases) were collected by population-based cancer registries affiliated with the CDC's National Program of Cancer Registries (NPCR) or the National Cancer Institute's Surveillance, Epidemiology, and End Results (SEER) program using medical records as the source of information for tumor and demographic characteristics [9, 10]. Both NPCR and SEER data were collected and reported using standard data items, uniform codes, and procedures as documented by the North American Association of Central Cancer Registries (NAACCR) [10]. We analyzed data from 46 population-based state cancer registries that met the United State Cancer Statistics (USCS) data publication standard for all years from 2001 to 2007 (http://www.cdc.gov/cancer/npcr/standards.htm). Data from Mississippi, Nevada, Tennessee, Virginia, and Washington DC were excluded because they did not meet these criteria for all study years. These 46 registries covered 93.5% of the US population. Cancer registries coded primary cancer site and histology according to criteria in the third edition of the International Classification of Diseases for Oncology (ICD-O-3) [11]. In this study, prostate cancer cases were identified by the ICD-O-3 site code C619 and behavior code 3. We excluded 16,510 autopsy only or death certificated only cases (1.3%) and 28,173 nonmicroscopically confirmed cases (2.0%). Because nearly 99% of prostate cancers are adenocarcinomas, we restricted our analyses to adenocarcinoma only, which we identified by the following ICD-O-3 histology codes: 8140, 8141, 8143, 8147, 8211, 8251, 8255, 8260, 8261, 8262, 8263, 8310, 8322, 8323, 8480, 8481, and 8550. As a result, we excluded additional 19,037 cases (1.2%) and the final study population was 1,310,373 cases. 

We stratified cases by age group (<40, 40–49, 50–59, 60–69, 70–79, or 80 years or above), race (white, black, Asian/Pacific Islander (API), American Indian/Alaska Native (AIAN), or unknown), Hispanic ethnicity, US Census region of residence (Northeast, Midwest, South, and West), and cancer stage and grade (see Table 1). For cancer registry purposes, we used SEER Summary staging instead of AJCC staging system in this study. Prostate cancer stage was based on SEER Summary Stage 2000 rules (http://seer.cancer.gov/tools/ssm/) for diagnosis years 2001–2003 and on Collaborative Stage rules (http://web.facs.org/cstage/schemalist.htm) for diagnosis years 2004–2007. To characterize cancer stage at diagnosis across all study years, we then combined these two staging systems and used three categories: no invasion beyond prostatic capsule (localized stage), invasion beyond prostatic capsule to adjacent organs or regional lymph nodes (regional stage), and invasion to distant organs or distant lymph nodes (distant stage). Prostate cancer grade was determined by Gleason score. Briefly, Gleason scores 2–4 were classified as well differentiated prostate cancer, 5 and 6 as moderately differentiated, and 7 and above as poorly differentiated. More information on Gleason's system can be found at http://training.seer.cancer.gov/prostate/abstract-code-stage/morphology.html. During 2001–2003, prostate cancers with a Gleason score 7 were coded in the cancer registries as moderately differentiated; however from 2004 to 2007, these cases were coded as poorly differentiated [12]. To minimize the impact of this coding change on our estimates of incidence rates during the study period, 2001–2007, we collapsed grade categories into 2 groups: well differentiated; and moderately/poorly/undifferentiated. However, we used a standard (unclasped) cancer grade variable in the trend analysis from 2004 to 2007 (see Table 3). 

We used annual population estimates as denominators to calculate incidence rates. All incidence rates were age-adjusted to the 2000 US standard population by the direct method. Annual percentage change (APC) is used to measure the changes in rates over time. We estimated APC of rates and the corresponding 95% confidence intervals (CIs). We considered trends statistically significant if the 95% CIs surrounding the APC did not include zero. Because only 700 prostate cancer cases were less than 40 years old from 2001 to 2007, we excluded this group when we examined age-specific incidence rates and trends by cancer stage and grade (see Table 2). We used SEER*Stat version 7.0.4 (http://www.seer.cancer.gov/seerstat) to conduct all analyses.

## 3. Results

As shown in Table 1, for all age combined, there were 1,310,373 newly diagnosed prostate cancer cases in the US from 2001 to 2007, with an age-adjusted incidence rate of 149.8 per 100,000. Of these cases, 82.5% were white, 12.3% were black, 1.8% were API, and 0.3% were AIAN. About 97.0% were men aged 50 years or older, 81.0% were localized, and 2.0% were well differentiated (Table 1).

Although the overall prostate cancer incidence remained stable from 2001 to 2007, there were statistically significant increases in the rate for men aged 0–39 years (APC = 6.6; 95% CI = 0.7, 12.7) and men aged 40–49 years (APC = 3.0; 95% CI = 0.6, 5.5), respectively. Statistically significant decreases in incidence were observed for men aged 70–79 years (APC = −2.3; 95% CI = −4.5, −0.1) and men aged 80 years and older (APC = −4.4; 95% CI = −5.8, −3.0), respectively. Significant declines in incidence also occurred among blacks (APC = −2.1; CI = −3.3, −0.9), AIANs (APC = −4.1; CI = −7.3, −0.7), APIs (APC = −3.2; CI = −4.8, −1.6), Hispanics (APC = −2.9; CI = −4.4, −1.4), and men residing in the West (APC = −1.8; CI = −3.3, −0.2). Incidence rates declined significantly over time for distant prostate cancer (APC = −3.1; CI = −4.4, −1.8) and well differentiated prostate cancer (APC = −26.0; CI = −27.3, −24.8), respectively, during the study years (Table 1).

As shown in Table 2, although the incidence of distant stage prostate cancer among men aged 40–49 years remained stable from 2001 to 2007, we observed statistically significant increases in the incidence of localized and regional diseases by 3.4% and 3.5% per year, respectively. Incidence of moderately/poorly/undifferentiated prostate cancer significantly increased by 3.8% per year from 2001 to 2007, accompanied by a significant decrease, 24.3% per year, among well differentiated cancer. For men aged 50–59 years, statistically significant declines in incidence occurred in both distant stage (APC = −1.7; CI = −2.7, −0.6) and well differentiated prostate cancers (APC = −24.2; CI = −27.5, −20.7). Likewise, significant declines were observed among men aged 60–69 years and men aged 70–79 years, respectively. For men aged 80 years or older, statistically significant declines in incidence occurred in each cancer stage and grade category. 

Table 3 shows cancer incidence rates and trends by stage and grade. For all age combined, there were 751,565 cases newly diagnosed from 2004 to 2007. About 82.0% of cases were localized. Moderately differentiated and poorly differentiated prostate cancers accounted for 49.0% and 46.4% of total cases, respectively. There was a statistically significant increase in the incidence of poorly differentiated prostate cancer by 6.8% per year from 2004 to 2007, specifically with increases of 8.0% and 6.1% per year among localized and regional prostate cancers, respectively. The incidences of well differentiated cancer declined significantly by 24.5% and 23.8% per year, respectively, among localized and regional prostate cancers. The incidences of moderately differentiated cancer significantly declined from 2004 to 2007 among regional (APC = −10.9; CI = −17.6, −3.8) and distant (APC = −18.6; CI = −28.2, −7.8) prostate cancers. We obtained similar results for prostate cancer incidence rates and trends by stage and grade when we restricted our analyses to men aged 50 years and above (data not shown). 

## 4. Discussion

There are several findings to emphasize from this study. First, these nationwide data show that although the overall prostate cancer incidence rate was stable from 2001 to 2007, rates significantly increased among men under age 50 years and decreased among men aged 70 years or older. Second, from 2004 to 2007, more than half of localized prostate cancers were well or moderately differentiated (Gleason scores ≤6). Last, poorly differentiated prostate cancer accounted for 42% and 76% of the localized and regional cancers, respectively. The incidence of poorly differentiated prostate cancer significantly increased for both localized and regional cancers from 2004 to 2007. 

Our findings that prostate cancer incidence trends differed dramatically between younger and older men are similar to a SEER study which showed that risks for being diagnosed with prostate cancer, for both blacks and whites, increased among men aged 30–49 years, but decreased among men aged 50 year and older from 2000 to 2007 [13]. It is well known that screening influences cancer incidence to some extent. The American Cancer Society recommends discussing prostate cancer screening with men who have at least a 10-year life expectancy at age 50 if they are at average risk [14]. Most major US medical organizations also recommend against prostate cancer screening among men who have a less than 10-year life expectancy [15–17]. Results form a National Health Interview Survey study showed that the prevalence of PSA screening among men aged 70 years or older was higher in 2005 than in 2000 [18]. Thus, prostate cancer screening might not account for the decreasing trend in prostate cancer incidence in this older population. Similarly, using Behavioral Risk Factor Surveillance System and NPCR/SEER data, we found discordance of prostate cancer incidence and screening among men aged 40–49 years [19]. Thus, screening alone might not explain the changes in prostate cancer incidence in certain age groups. Additional research is necessary on changes of physicians' and patients' knowledge and perspectives of prostate cancer screening, uptake of age-stratified PSA threshold for a diagnostic biopsy, and changes in environmental and behavioral risk factors of prostate cancer to better understand these discrepancies. 

In 2002, Stephenson examined 1973–1997 SEER data and found that, after the introduction of PSA testing, localized and regional diseases substantially increased with a dramatic decrease in distant disease [7]. We found that from 2001 to 2007, incidence of distant stage prostate cancer declined only among men aged 50 or older, but not men aged 40–49 years. The reason for this age-specific decline is possibly because prostate cancer screening guidelines from major medical organizations during that time recommended men at average risk have a screening test at age 50. Our study demonstrated a dramatic decline in incidence of well differentiated cancers from 2001 to 2007 in each age group. One possible explanation for this decline is shift toward upgrading cancer using the Gleason Scoring system [6]. Recently, pathologists have tended to assign relatively high Gleason scores to biopsy specimens of prostate in order to align more closely with scores generated from reviews of the entire surgical specimen [20]. Another possible explanation is that, in 2005, the International Society of Urologic Pathologists (ISUP) and the World Health Organization recommended pathologists report all higher tertiary grade components of the cancer as part of a Gleason score [21].

Age at diagnosis, cancer stage, and grade are important factors in determining the prostate cancer treatment regimens that influence health outcomes. For instance, Lin et al. found that men aged 35–44 years with high-grade prostate cancer had worse overall survival and disease-specific survival compared with older men [22]. As shown in our study, over 80% of prostate cancer cases were localized stage. Treatment modalities for the localized prostate cancer markedly vary by cancer grade. According to the National Comprehensive Cancer Network (NCCN) guideline (http://www.nccn.com/cancer-guidelines.html#prostate), patients with a localized, low recurrence risk cancer (stage T1-T2a, Gleason scores ≤6, and PSA < 10 ng/mL) could be managed with active surveillance or surgery or radiation alone. In particular, patients with a less than 20-year life expectancy and a very low recurrence risk prostate cancer (stage T1c), identified by needle biopsy after an elevated PSA, may be more appropriately followed without immediate intervention such as active surveillence. Our study shows that, from 2004–2007, about 56% of localized prostate cancers (stage T1 or T2) were well or moderately differentiated (Gleason scores ≤6). These cancers were more likely to be categorized as low recurrence risk if they were in stage T1or T2a (according to NCCN, stage T2b and T2c were in intermediate risk group). Using data from the Cancer of the Prostate Strategic Urologic Research Endeavor (CaPSUPE) registry, Cooperberg et al. found that only 9% of men with localized low recurrence risk prostate cancer did not undergo immediate treatment [23]. Thus, non preferability of active surveillance among localized low recurrence risk prostate cancer patients need to be further investigated. 

Interestingly, we found that up to 43% of localized prostate cancers were poorly or undifferentiated and that the incidence of poorly differentiated cancer rose 8.0% and 6.1% per year from 2004 to 2007 for localized and regional prostate cancers, respectively. These increasing trends may be partially attributed to the acceptance and implementation of the 2005 ISUP recommendation, which called for reporting higher tertiary Gleason patterns and reporting different Gleason grades in multiple needle biopsies [21]. However, possible effects of new environmental or behavioral exposures to incur more aggressive prostate cancer cannot be completely ruled out. Continued monitoring of the pathological pattern in prostate cancer is greatly needed to better understand the increasing trend of the poorly differentiated cancer, especially with the US Preventive Service Task Force's draft recommendation against prostate cancer screening for men of all ages [24].

Using cancer registry data, covering over 93% of the US population enables our study to have broad generalizability. However, this study is subject to at least three limitations. First, because of changes in the grading system using Gleason score, we had to combine moderately and poorly differentiated, and undifferentiated tumors into one category to achieve a complete trend analysis from 2001 to 2007. Second, cancer registry collected the “best available grade.” Therefore, for men elect nonsurgically therapy, the Gleason score, which was based on biopsy, may be underrated compared with men treated surgically sooner after the diagnosis. However, urological pathologist tends to assign a higher Gleason score to biopsy specimen to address this issue. Our estimates of poorly differentiated cancer (42%) were conservative given the issue of underestimation of Gleason score. Third, we documented a substantial number of cancers that were unstaged or had an unknown grade. This problem diminished with time indicating better data quality in more recent years. Last, although NPCR and SEER data are the most geographically comprehensive data available, not all states were included in the analysis (from 84% in the South to 100% in the Northeast). 

In conclusion, this study shows opposing trends of prostate cancer incidence among younger and older men. Significant decreases in well differentiated and distant stage prostate cancers might suggest effects of PSA testing in the post-PSA-era; however, prostate cancer screening alone cannot explain the changes in prostate cancer trends. Most of the localized prostate cancers were low grade, suggesting active surveillance as a possible treatment option. Given the large proportion of poorly differentiated disease among localized prostate cancers and its increasing trend in more recent years, continued monitoring of prostate cancer incidence and trends by demographic and tumor characteristics is warranted, especially with US Preventive Service Task Force's recommendation against prostate cancer screening for American men.

## Figures and Tables

**Table 1:** Counts, incidence rates, and annual percentage changes in rates for prostate cancer by demographic and clinical characteristics, US men, 2001–2007.

	Count	%	Rate^1^	95% CI		APC	95% CI	
Total^2^	1,310,373	100.0	149.8	149.6	150.1	−1.6	−3.8	0.7
Age								
<40	700	0.1	0.1	0.1	0.2	6.6	0.7	12.7
40–49	35,648	2.7	23.9	23.7	24.2	3.0	0.6	5.5
50–59	257,120	19.6	221.0	220.2	221.9	0.0	−2.4	2.5
60–69	475,625	36.3	693.7	691.7	695.6	−0.8	−3.3	1.7
70–79	409,460	31.2	897.3	894.6	900.1	−2.3	−4.5	−0.1
80+	131,820	10.1	541.6	538.6	544.5	−4.4	−5.8	−3.0
Race								
White	1,080,742	82.5	141.2	140.9	141.5	−1.9	−4.2	0.4
Black	161,158	12.3	218.4	217.3	219.5	−2.1	−3.3	−0.9
AIAN	4,329	0.3	74.0	71.7	76.4	−4.1	−7.3	−0.7
API	23,018	1.8	79.5	78.4	80.5	−3.2	−4.8	−1.6
Unknown^3^	41,126	3.1	~	~	~	~	~	~
Ethnicity^4^								
Non-Spanish-Hispanic-Latino	1,236,978	94.4	152.3	152.0	152.5	−1.4	−3.7	0.9
Spanish-Hispanic-Latino	73,395	5.6	120.7	119.8	121.6	−2.9	−4.4	−1.4
Region								
Northeast	297,140	22.7	162.2	161.6	162.8	−1.4	−4.7	2.1
Midwest	322,544	24.6	152.2	151.7	152.8	−1.7	−3.6	0.1
South	412,862	31.5	143.8	143.4	144.3	−1.3	−3.7	1.2
West	277,827	21.2	144.3	143.8	144.9	−1.8	−3.3	−0.2
Stage^5^								
Localized	1,061,748	81.0	121.4	121.1	121.6	−1.0	−3.2	1.3
Regional	126,142	9.6	13.6	13.6	13.7	0.2	−1.8	2.3
Distant	41,342	3.2	5.0	4.9	5.0	−3.1	−4.4	−1.8
Unstaged	81,116	6.2	9.8	9.7	9.9	−9.4	−13.6	−5.0
Grade								
Well differentiated	25,558	2.0	3.0	3.0	3.0	−26.0	−27.3	−24.8
Moder/poor/undifferentiated	1,233,810	94.2	140.7	140.5	141.0	−0.5	−2.4	1.5
Unknown	51,005	3.9	6.1	6.1	6.2	−12.4	−18.2	−6.3

AIAN: American Indian/Alaska Native, APC: annual percentage change, API: Asian/Pacific Islander, CI: confidence interval, moder/poor/undifferentiated: moderately, poorly, or undifferentiated.

^1^Rates are per 100,000 men and are age-adjusted to the 2000 US standard population.

^2^Data are from 46 population-based cancer registries that participate in the National Program of Cancer Registries (NPCR) and/or the Surveillance, Epidemiology, and End Results (SEER) Program and meet high quality data criteria. These registries cover 93.5% US population for 2001–2007.

^3^Unknown race and race groups other than white, black, AIAN, and API are not listed but included in the total case count.

^4^Hispanic origin is not mutually exclusive from race categories (white, black, AIAN, and API).

^5^Sum of counts is less than total due to missing information on stage.

~ indicates that the denominator for the rate was unknown.

**Table 2:** Incidence rates and annual percentage changes in rates for prostate cancer by age, stage, and grade, US men over 40 years, 2001–2007.

	Count^1^	%	Rate^2^	95% CI		APC	95% CI	
40–49 years								
Stage^3^								
Localized	27,959	78.4	18.8	18.6	19.0	3.4	0.9	5.9
Regional	5,360	15.0	3.6	3.5	3.7	3.5	0.8	6.3
Distant	1,048	2.9	0.7	0.7	0.7	1.5	−1.2	4.3
Unstaged	1,279	3.6	0.9	0.8	0.9	−5.2	−12.5	2.7
Grade								
Well differentiated, grade I	512	1.4	0.3	0.3	0.4	−24.3	−31.3	−16.6
Moder/poor/undifferentiated	34,161	95.8	22.9	22.7	23.2	3.8	1.7	5.9
Unknown	975	2.7	0.7	0.6	0.7	−6.8	−17.5	5.3
50–59 years								
Stage^3^								
Localized	205,439	79.9	176.6	175.8	177.3	0.3	−2.3	3.1
Regional	36,709	14.3	31.6	31.3	31.9	0.8	−1.2	2.8
Distant	5,542	2.2	4.8	4.6	4.9	−1.7	−2.7	−0.6
Unstaged	9,423	3.7	8.1	7.9	8.3	−7.9	−11.9	−3.7
Grade								
Well differentiated, grade I	3,924	1.5	3.4	3.3	3.5	−24.2	−27.5	−20.7
Moder/poor/undifferentiated	246,555	95.9	211.9	211.1	212.8	0.9	−1.3	3.1
Unknown	6,641	2.6	5.7	5.6	5.9	−12.9	−21.8	−3.1
60–69 years								
Stage^3^								
Localized	386,583	81.3	564.1	562.3	565.9	−0.7	−3.3	2.0
Regional	55,990	11.8	81.2	80.5	81.9	0.8	−1.5	3.1
Distant	10,917	2.3	15.9	15.6	16.2	−2.0	−3.8	−0.1
Unstaged	22,120	4.7	32.4	32.0	32.9	−7.0	−11.1	−2.8
Grade								
Well differentiated, grade I	8,600	1.8	12.6	12.3	12.9	−26.1	−27.7	−24.6
Moder/poor/undifferentiated	452,138	95.1	659.3	657.4	661.2	0.1	−2.1	2.4
Unknown	14,887	3.1	21.8	21.4	22.1	−12.7	−19.7	−5.2
70–79 years								
Stage^3^								
Localized	342,813	83.7	751.2	748.7	753.7	−1.7	−3.8	0.4
Regional	22,923	5.6	49.9	49.3	50.6	−1.2	−3.9	1.6
Distant	13,226	3.2	29.1	28.6	29.6	−3.5	−5.5	−1.5
Unstaged	30,498	7.4	67.1	66.3	67.8	−9.0	−13.3	−4.6
Grade								
Well differentiated, grade I	9,157	2.2	20.1	19.7	20.5	−26.3	−27.8	−24.7
Moder/poor/undifferentiated	382,224	93.3	837.5	834.8	840.2	−1.2	−3.1	0.8
Unknown	18,079	4.4	39.7	39.1	40.3	−13.4	−18.5	−7.9
80+ years								
Stage^3^								
Localized	98,414	74.7	402.5	399.9	405.0	−2.7	−4.0	−1.4
Regional	5,080	3.9	21.0	20.5	21.6	−4.8	−7.3	−2.2
Distant	10,577	8.0	44.4	43.5	45.2	−4.6	−6.2	−2.8
Unstaged	17,748	13.5	73.7	72.6	74.8	−13.1	−17.9	−8.0
Grade								
Well differentiated; grade I	3,354	2.5	13.8	13.3	14.3	−27.0	−28.1	−25.8
Moder/poor/undifferentiated	118,070	89.6	484.3	481.5	487.1	−3.1	−4.3	−2.0
Unknown	10,396	7.9	43.5	42.6	44.3	−10.8	−14.9	−6.4

APC: annual percentage change, CI: confidence interval, moder/poor/undifferentiated: moderately, poorly, or undifferentiated.

^1^Data are from 46 population-based cancer registries that participate in the National Program of Cancer Registries (NPCR) and/or the Surveillance, Epidemiology, and End Results (SEER) Program and meet high quality data criteria. These registries cover 93.5% US population for 2004–2007.

^2^Rates are per 100,000 men and are age-adjusted to the 2000 US standard population.

^3^Sum of counts is less than total due to missing information on stage.

**Table 3:** Incidence rates and annual percentage changes in rates for prostate cancer by cancer stage and grade, US men, 2004–2007.

	Count	%	Rate^1^	95% CI		APC	95% CI	
Total^2^	751,565	100.0	145.3	144.9	145.6	1.9	−3.4	7.5
Stage^3^								
Localized	613,170	81.6	118.7	118.4	119.0	2.3	−3.2	8.1
Regional	75,412	10.0	13.7	13.6	13.8	1.8	−4.5	8.6
Distant	23,455	3.1	4.8	4.7	4.9	−3.2	−8.0	1.8
Unstaged	39,503	5.3	8.1	8.0	8.2	−0.2	−1.6	1.1
Grade								
Well differentiated	8,618	1.1	1.7	1.7	1.7	−25.5	−29.5	−21.2
Moderately differentiated	368,482	49.0	70.3	70.0	70.5	−1.8	−6.6	3.2
Poorly differentiated	348,426	46.4	68.0	67.8	68.2	6.8	1.3	12.7
Undifferentiated, anaplastic	3,212	0.4	0.6	0.6	0.7	1.0	−7.3	9.9
Unknown	22,827	3.0	4.7	4.6	4.7	1.1	−9.3	12.6
Localized								
Well differentiated	7,714	1.3	1.5	1.5	1.6	−24.5	−26.4	−22.5
Moderately differentiated	333,625	54.4	63.7	63.4	63.9	−1.3	−6.1	3.7
Poorly differentiated	259,719	42.4	51.1	50.9	51.3	8.0	2.0	14.3
Undifferentiated, anaplastic	1,939	0.3	0.4	0.4	0.4	6.3	−5.5	19.7
Unknown	10,173	1.7	2.0	2.0	2.1	0.2	−16.0	19.6
Regional								
Well differentiated	210	0.3	0.0	0.0	0.0	−23.8	−42.3	0.7
Moderately differentiated	16,449	21.8	2.9	2.9	3.0	−10.9	−17.6	−3.8
Poorly differentiated	57,506	76.3	10.5	10.4	10.6	6.1	0.6	11.8
Undifferentiated, anaplastic	656	0.9	0.1	0.1	0.1	−6.7	−21.4	10.7
Unknown	591	0.8	0.1	0.1	0.1	−6.8	−32.1	28.0
Distant								
Well differentiated	95	0.4	0.0	0.0	0.0	−13.7	−46.2	38.3
Moderately differentiated	1,903	8.1	0.4	0.4	0.4	−18.6	−28.2	−7.8
Poorly differentiated	17,376	74.1	3.5	3.5	3.6	−1.6	−6.6	3.7
Undifferentiated, anaplastic	451	1.9	0.1	0.1	0.1	−8.2	−30.0	20.5
Unknown	3,630	15.5	0.8	0.7	0.8	−1.4	−10.2	8.4
Unstaged								
Well differentiated	599	1.5	0.1	0.1	0.1	−38.7	−60.4	−4.9
Moderately differentiated	16,488	41.7	3.3	3.2	3.3	−1.0	−2.4	0.4
Poorly differentiated	13,818	35.0	2.9	2.8	2.9	0.4	−2.7	3.7
Undifferentiated, anaplastic	166	0.4	0.0	0.0	0.0	−5.0	−35.0	38.8
Unknown	8,432	21.3	1.8	1.7	1.8	3.6	−5.4	13.4

APC: annual percentage change, CI: confidence interval.

^1^Rates are per 100,000 men and are age-adjusted to the 2000 US standard population.

^2^Data are from 46 population-based cancer registries that participate in the National Program of Cancer Registries (NPCR) and/or the Surveillance, Epidemiology, and End Results (SEER) Program and meet high quality data criteria. These registries cover 93.5% US population for 2004–2007.

^3^Sum of counts is less than total due to missing information on stage.

## Abbreviations

AIAN: American Indian/Alaska Native
API: Asian/Pacific Islander
CI: Confidence interval
ICD-O-3: International Classification of Diseases for Oncology
NCCN: National Comprehensive Cancer Network
NPCR: National Program of Cancer Registries
PSA: Prostate-specific antigen
SEER: Surveillance, Epidemiology, and End Results.

## References

[1] U.S. Cancer Statistics Working Group (2010). *United States Cancer Statistics: 1999–2006 Incidence and Mortality Web-Based Report*.

[2] Stanford JL, Stephenson RA, Coyle LM (1999). Prostate Cancer Trends 1973–1995, SEER Program, National Cancer Institute. *NIH*.

[3] Welch HG, Albertsen PC (2009). Prostate cancer diagnosis and treatment after the introduction of prostate-specific antigen screening: 1986–2005. *Journal of the National Cancer Institute*.

[4] Kohler BA, Ward E, McCarthy BJ (2011). Annual report to the nation on the status of cancer, 1975–2007, featuring tumors of the brain and other nervous system. *Journal of the National Cancer Institute*.

[5] Shao YH, Demissie K, Shih W (2009). Contemporary risk profile of prostate cancer in the United States. *Journal of the National Cancer Institute*.

[6] Jani AB, Johnstone PAS, Liauw SL, Master VA, Brawley OW (2008). Age and grade trends in prostate cancer (1974–2003): a surveillance, epidemiology, and end results registry analysis. *American Journal of Clinical Oncology*.

[7] Stephenson RA (2002). Prostate cancer trends in the era of prostate-specific antigen an update of incidence, mortality, and clinical factors from the SEER database. *Urologic Clinics of North America*.

[8] Jani AB, Hellman S (2003). Early prostate cancer: clinical decision-making. *The Lancet*.

[9] Hankey BF, Ries LA, Edwards BK (1999). The surveillance, epidemiology, and end results program: a national resource. *Cancer Epidemiology Biomarkers and Prevention*.

[10] Wingo PA, Jamison PM, Hiatt RA (2003). Building the infrastructure for nationwide cancer surveillance and control—a comparison between The National Program of Cancer Registries (NPCR) and The Surveillance, Epidemiology, and End Results (SEER) Program (United States). *Cancer Causes and Control*.

[11] Fritz A, Percy C, Jack A (2000). *International Classification of Disease for Oncology*.

[12] Johnson CH (2004). SEER Program Coding and Staging Manual 2004, Revision 1. National Cancer Institute. *NIH*.

[13] Merrill RM, Sloan A (2012). Risk-adjusted incidence rates for prostate cancer in the United States. *Prostate*.

[14] Wolf AMD, Wender RC, Etzioni RB (2010). American Cancer Society guideline for the early detection of prostate cancer: update 2010. *CA Cancer Journal for Clinicians*.

[15] (1997). Screening for prostate cancer. American College of Physicians. *Annals of Internal Medicine*.

[16] (2000). Prostate-specific antigen (PSA) best practice policy. American Urological Association (AUA). *Oncology*.

[17] Lim LS, Sherin K (2008). Screening for prostate cancer in U.S. men ACPM position statement on preventive practice. *American Journal of Preventive Medicine*.

[18] Drazer MW, Huo D, Schonberg MA, Razmaria A, Eggener SE (2011). Population-based patterns and predictors of prostate-specific antigen screening among older men in the United States. *Journal of Clinical Oncology*.

[19] Li J, German R, King J (2012). Recent trends in prostate cancer testing and incidence among men under age of 50. *Cancer Epidemiology*.

[20] Albertsen PC, Hanley JAH, Barrows GH (2005). Prostate cancer and the Will Rogers phenomenon. *Journal of the National Cancer Institute*.

[21] Epstein JI, Allsbrook WC, Amin MB (2005). The 2005 International Society of Urological Pathology (ISUP) consensus conference on Gleason grading of prostatic carcinoma. *American Journal of Surgical Pathology*.

[22] Lin DW, Porter M, Montgomery B (2009). Treatment and survival outcomes in young men diagnosed with prostate cancer: a population-based cohort study. *Cancer*.

[23] Cooperberg MR, Broering JM, Carroll PR (2010). Time trends and local variation in primary treatment of localized prostate cancer. *Journal of Clinical Oncology*.

[24] Chou R, Croswell JM, Dana T (2011). Screening for prostate cancer: a review of the evidence for the U.S. Preventive Services Task Force. *Annals of Internal Medicine*.

